# Is It Homœopathy or Isopathy?

**Published:** 1891-12

**Authors:** Samuel Swan


NOTE: The proof of the foregoing article having been shown to Dr. Swan, he wrote the following reply:

IS IT HOMŒOPATHY OR ISOPATHY?

Hahnemann, on page 196, *Chronic Diseases*, says, "I call Psorin a homœopathic antipsoric, because if the *preparation* (potentization) of Psorin *did not alter its nature to that of a homœopathic remedy*, it never could have any effect upon an organism *tainted with that same identical virus.*" (Italics are mine.) What is said of Psorin is equally applicable to all morbose products. Experience proves that the symptoms of a disease are a virtual proving. A disease may have more than one morbose product, but the virus that caused the disease lies in the product, whether it be in the pus or gall or blood. Dr. Swan is satisfied with his belief in the symptoms of a disease being a proving, and those who do not so believe should prove them themselves. One thing is a fact, these morbose products potentized do cure. They are either isopathic as some contend or homœopathic, but they cure under either name. Hahnemann says they must be homœopathic or they would not cure. S. SWAN.

